# Anatomy- versus Sensitivity-Based Loci Preselection in Detecting USH2A-Retinopathy Microperimetric Progression

**DOI:** 10.1016/j.xops.2025.101018

**Published:** 2025-11-24

**Authors:** Jason Charng, David Alonso-Caneiro, Tina M. Lamey, Jennifer A. Thompson, Jeremiah K.H. Lim, Elaine Ong, Terri L. McLaren, Fred K. Chen

**Affiliations:** 1 Centre of Ophthalmology and Visual Science (incorporating Lions Eye Institute), The University of Western Australia, Western Australia; 2 Department of Optometry and Vision Science, School of Health and Clinical Sciences, The University of Western Australia, Perth, Australia; 3 School of Science, Technology and Engineering, University of Sunshine Coast, Petrie, Queensland, Australia; 4 Contact Lens and Visual Optics Laboratory, Centre for Vision and Eye Research, School of Optometry and Vision Science, Queensland University of Technology (QUT), Kelvin Grove, Australia; 5 Australian Inherited Retinal Disease Registry and DNA Bank, Department of Medical Technology and Physics, Sir Charles Gairdner Hospital, Nedlands, Western Australia, Australia; 6 Ophthalmology, Department of Surgery, University of Melbourne, East Melbourne, Victoria, Australia; 7 Department of Ophthalmology, Royal Perth Hospital, Perth, Western Australia; 8 Royal Victorian Eye and Ear Hospital, East Melbourne, Victoria, Australia

**Keywords:** Autofluorescence imaging, Microperimetry, Retinitis pigmentosa, Trial end point, *USH2A*

## Abstract

**Purpose:**

To compare microperimetry progression rate in *USH2A-*retinopathy using prespecified points based on fundus autofluorescence coregistration with loci preselected based on retinal sensitivity profile.

**Design:**

Cohort longitudinal study.

**Subjects:**

Seventeen eyes from 17 patients with biallelic pathogenic variants in *USH2A* gene.

**Methods:**

Microperimetry was recorded using 10-2 grid. The grid was partitioned into 68 2° × 2° nonoverlapping squares, representing the retinal coverage of each locus. Four metrics were defined at baseline: (1) mean macular sensitivity (MMS): average sensitivity of all loci; (2) edge of scotoma sensitivity (ESS): average sensitivity of all loci adjacent to a scotomatous loci at baseline; (3) modified Rate of Progression in *USH2A*-related Retinal Degeneration study-defined functional transitional point (mFTP): selection based on ranking of the proportion peripheral adjacent loci that showed ≥7 decibel (dB) decrease; and (4) hyperautofluorescent ring sensitivity (HRS): average sensitivity of stimulus squares which the hyperautofluorescent ring boundary transects into. Trend-based progression rates (gradient from linear regression) were compared between these metrics, and event-based analysis of the US Food and Drug Administration-defined clinically significant change in visual field (mean change of ≥7 dB across ≥5 prespecified loci).

**Main Outcome Measures:**

Trend- and event-based measures in MMS, ESS, mFTP, and HRS.

**Results:**

Seventeen patients (median age 37.0 years) had mean baseline values of 9.7 dB, 9.2 dB, 17.9 dB, and 13.1 dB for MMS, ESS, mFTP, and HRS, respectively. Using all longitudinal data (mean follow-up 4.0 years), trend analysis showed mFTP progression rate (–1.53 ± 1.37 dB/year) was significantly faster than MMS (–0.51 ± 0.63 dB/year) and ESS (–1.11 ± 1.23 dB/year) but similar to HRS (–1.29 ± 1.41 dB/year). Edge of scotoma sensitivity was more prone to floor effect and had lower baseline sensitivity than mFTP and HRS. In event-based analysis, the proportion of eyes that demonstrated clinically significant mean change was similar between ESS (2-year 36.4%, overall 45.5%), mFTP (2-year 33.3%, overall 43.8%), and HRS (2-year 28.5%, overall 42.9%) but noticeable less in MMS (2-year 13.3%, overall 12.5%).

**Conclusions:**

Hyperautofluorescent ring sensitivity and mFTP showed comparable performance in both trend- and event-based analyses, superior to that of MMS and ESS. Additional advantage of mFTP is inclusion of patients without the autofluorescent ring.

**Financial Disclosure(s):**

Proprietary or commercial disclosure may be found in the Footnotes and Disclosures at the end of this article.

Usherin, encoded by the *USH2A* gene,^1^ is a critical structural protein localized in the connecting cilium of photoreceptors^2^ and the stereocilia ankle links in cochlear hair cells.^3^ Mutation in the *USH2A* gene can lead to isolated retinitis pigmentosa (RP) or syndromic RP with associated hearing loss.^4^ It has been shown that biallelic *USH2A* pathogenic variants are the leading cause of autosomal-recessive RP (∼20%)^5^^,^^6^ and has been estimated to account for ∼8% of inherited retinal disease.^7^^,^^8^ Given the relatively high prevalence of *USH2A*-retinopathy, detailed functional assessment is essential for clinical management. Furthermore, with several gene therapy trials targeting *USH2A*-retinopathy in the pipeline (Théa Sepofarsen, *USH2A* gene editing via CRISPR^9^), appropriate functional outcome measures are required for trial entry as well as endpoints.

As visual acuity is a poor surrogate measure for photoreceptor loss, microperimetry is regarded as a key functional measure in the inherited retinal disease clinic and clinical trials. Microperimetry measures retinal sensitivity while compensating for eye movements during the test, allowing the projection of spatially precise stimuli onto the retinal plane. Furthermore, retinal landmarks from the baseline examination are used in subsequent tests to track retinal sensitivity at the same locations across time. The MAIA (Macular Integrity Assessment; CenterVue) device is a commercially available confocal scanning laser ophthalmoscope-based microperimeter with robust intersession agreement^10^ and coefficient of repeatability.^11^ The device has been widely used in routine retinal clinical care^12^^,^^13^ and to track retinal function in clinical trials.^14^^,^^15^ Several microperimetry parameters have been analyzed to estimate retinal function, including pointwise sensitivity,^16^^,^^17^ mean macular sensitivity (MMS),^18^^,^^19^ and sensitivity of nonscotomatous points.^11^^,^^20^ Additionally, it has been demonstrated that retinal sensitivity in loci surrounding scotomatous points (edge of scotoma sensitivity [ESS]) show faster progression rate than MMS in age-related macular degeneration,^21^ Stargardt disease,^22^ and *USH2A*-retinopathy.^20^ Recently, the Rate of Progression in *USH2A*-related Retinal Degeneration (RUSH2A) group proposed the metric Function Transition Point (FTP), which selected loci based on a ranking of ≥7 decibel (dB) change compared with adjacent loci at baseline.^23^ One posits that the majority of the FTP loci will be located next to a scotomatous locus. However, it has been shown that microperimetric test-retest repeatability is significantly higher at the border of deep scotoma than normal retina.^24^ Hence, the prespecified selection of ESS and FTP for progression analyses may be confounded by a greater test-retest variability. Another limitation of FTP is that it cannot be applied to the commonly used 10-2 MAIA test grid as the FTP was defined on the 89-loci pattern used in the RUSH2A studies. A modified FTP (mFTP) selection protocol is needed for applying this strategy to analyze the large number of 68-loci 10-2 MAIA data that have already been collected by numerous clinical studies. One approach to decrease variability of functional measures is to colocalize the data to anatomic findings. In RP, including eyes with *USH2A*-retinopathy, a classic finding is presence of the hyperautofluorescent ring (HAR) at the posterior pole in autofluorescence (AF) imaging.^25^ In short-wavelength AF, it has been shown that the hyperautofluorescent signal corresponds to abnormal accumulation in lipofuscin.^26^ The ring boundary marks structural and functional transition zone between healthy and diseased retina^27^, ^28^, ^29^, ^30^, ^31^ and, importantly, contracts over time.^32^, ^33^, ^34^ Hence, retinal sensitivity at the HAR boundary, or HAR sensitivity (HRS), may be a more robust metric for progression analysis.

In this study, we examined the progression rates of microperimetry parameters in *USH2A*-retinopathy. In particular, sensitivity-based metrics such as MMS, ESS, and mFTP progression rates were compared with progression in prespecified HRS.

## Methods

Ethics approval was obtained from the human ethics committees at the University of Western Australia (2021/ET000151) and Sir Charles Gairdner Hospital (RGS04985), Perth, Western Australia, Australia. Written informed consent was obtained from all participants for their data to be used for research purposes and tenets of the Declaration of Helsinki were adhered to.

### Patient Selection

The Western Australian Retinal Degeneration and the Australian Inherited Retinal Disease Registry databases were interrogated for patients with biallelic pathogenic or likely pathogenic (PLP) variants in the *USH2A* gene. In this prospective longitudinal study, patients were examined and measured every 6 months for 5 years. In addition, only patients with serial microperimetry and a concurrent short-wavelength fundus AF (FAF) data at baseline were included in the study.

### Genetic Diagnosis

Patient DNA was analyzed using various clinical-grade next-generation sequencing panels targeting known retinal dystrophy genes, with complete coverage of all exons, flanking intronic regions, and reported deep intronic variants. Sequencing data were aligned to genomic build GRCh37. To confirm biallelism of candidate *USH2A* variants, targeted Sanger sequencing was performed using DNA from appropriate family members. *USH2A* variants were confirmed to be in *trans* configuration for all patients by family segregation analysis. *USH2A* variants were annotated using cDNA reference sequence, NM_206933.2 to NM_206933.4; USH2A protein RefSeq, NP_996816.2 to NP_996816.4 (Molecular Vision Laboratory). The pathogenicity of biallelic variants was assessed by the AIRDR^35^ in accordance with recommendations by the American College of Medical Genetics and Genomics,^36^ and the ClinGen Sequence Variation Interpretation Working Group (https://clinicalgenome.org/working-groups/sequence-variant-interpretation/).

### Autofluorescence Imaging Protocol

Fundus AF images were acquired with a confocal scanning laser ophthalmoscope (cSLO; Heidelberg Spectralis HRA, Heidelberg Engineering), as previously described.^37^ Briefly, short-wavelength AF images (488 nm excitation, >500 nm emission) were obtained with a 30° × 30° acquisition window in high resolution mode. The manufacturer’s image averaging function (Automatic Real Time) was enabled during acquisition to improve image quality. During image acquisition ≥50 frames were averaged if 100 frames could not be tolerated.

### Microperimetry Protocol

Macular Integrity Assessment microperimetry was performed by trained ophthalmic assistants in a completely darkened room. Eyes were dilated (tropicamide 0.5% and phenylephrine 2.5%) before testing and patients sat in the darkened room for 2 to 3 minutes before commencing the test.

Test grid consisted of 68 loci arranged in a square array, centered on the fovea. The grid spanned 18° in diameter and adjacent test loci were located 1°, 3°, 5°, 7°, and 9° from the vertical or horizontal meridian, akin to the 10-2 grid in Humphrey Field Analysers. Goldmann III achromatic stimuli with a 200-ms duration were displayed on a white background (1.27 cd/m^2^) via a 4-2 test strategy. The dynamic range of the differential stimulus luminance was 0.08 to 317.04 cd.m^–2^ (36 to 0 dB). For analysis, a –1 dB value was assigned to a locus if the stimulus could not be detected at the highest luminance.

Microperimetry data exported included raw retinal sensitivity values at each locus. Additionally, the device also generated a cSLO image of the posterior pole with the test grid superimposed, which was also extracted for image registration.

### Image Segmentation and Registration

Only eyes with a complete HAR were included in the study. A HAR was considered incomplete if parafoveal retinal pigment epithelium atrophy surrounded or eroded into the HAR, thus interrupting the smooth boundary. Hyperautofluorescent rings were first delineated using a deep-learning algorithm followed by manual supervision of the segmentation results, and correction if necessary.^32^

The segmented AF images were exported in bitmap format and registered to the MAIA cSLO image using a customized MATLAB program (version: 24.1.0 [R2024a], The MathWorks Inc) by manually selecting retinal landmarks common to both images. For both images, the size to pixel information was encoded into the program (MAIA image: 36° × 36°, 1024 × 1024 pixels; short-wavelength AF: 30° × 30°, 768 × 768 pixels). The program then outputs ring boundary in Cartesian format (in degrees), relative to the center of the MAIA grid and appropriately scaled for size.

### Data Analysis

One eye per patient was selected for analysis, with left eye chosen as 1 patient had no longitudinal data in the right eye. For each eye, baseline was defined as the first visit with concurrent HAR and MAIA data. Only MAIA data recorded with the “Follow-up” mode were included in longitudinal analysis. To be eligible for longitudinal analysis, each eye must have a minimum of 2 years longitudinal data and ≥3 microperimetry tests. Longitudinal analysis was conducted for all available data. Additionally, a subanalysis was performed for the first 2 years of longitudinal data. The 2-year timeframe was chosen to simulate the duration of a gene therapy clinical trial.

Four parameters from raw retinal sensitivity values were extracted at baseline. First, MMS refers to the average sensitivity values across all 68 loci in the test grid, including those with –1 dB output. Second, ESS is the average value of any loci adjacent to any scotomatous (i.e., –1 dB) locus, as previously proposed.^21^ Third, mFTP loci were preselected based on a ranking of the proportion of ≥7 dB decrease in the adjacent loci.^23^ However, the test grid used in the RUSH2A study differs to that of the 10-2 grid in the current work. Hence a new schematic of the pathway for selecting mFTP was devised (Fig. S1, available at www.ophthalmologyscience.org), with the number of adjacent loci connected to a locus ranging from 2 to 5. For each locus, the proportion of adjacent loci that showed a decrease ≥7 dB (i.e., a decline of ≥7 dB) was calculated (Excel spreadsheet available in Appendix S1, available at www.ophthalmologyscience.org). To increase comparability to the RUSH2A data, each mFTP locus has retinal sensitivity of ≥8 dB at baseline. A locus was chosen for mFTP analysis if the proportion was 100%. In the case where the number of 100% proportion loci was <5, points with next highest proportion were included until ≥5 loci were selected. Fourth, HRS was defined as the average sensitivity value of loci found near the HAR boundary. More specifically, the 68-loci grid was represented by 68 nonoverlapping 2° × 2° squares, with the center of the square coinciding with center of the Goldmann III stimulus. A locus is included into HRS analysis if the ring boundary transects into its corresponding square after FAF and MAIA output coregistration. Note that ESS, mFTP, and HRS loci were defined at baseline and the same loci were used throughout the longitudinal analysis.

Unless otherwise specified, group data are described by mean and standard deviation. In longitudinal trend analysis, linear regression was conducted for MMS, ESS, mFTP, and HRS for each eye across time and the slope values were extracted as an estimate of progression. There was no minimum baseline loci requirement in the longitudinal trend analyses. In HRS progression analyses, loci with –1 dB at baseline were excluded. In both 2-year and overall longitudinal data, a repeated measures analysis of variance (ANOVA) was conducted to compare against MMS, ESS, mFTP, and HRS rates across all eyes for each follow-up period. In the case of an overall significant difference in means, post hoc pairwise comparison with Bonferroni correction was used to identify significant difference between microperimeter metrics. Each *P* value in pairwise comparison has been corrected for multiple comparisons, with *P* < 0.05 indicating statistical significance between groups. The US Food and Drug Administration (FDA) has defined a mean change ≥7 dB across ≥5 prespecified loci as clinically meaningful change in microperimetry event analysis.^38^^,^^39^ Hence, an event-based analysis was conducted in eyes with ≥5 prespecified loci within the ESS, mFTP, and HRS metrics that showed a sensitivity ≥8 dB at baseline to ensure compliance with the FDA guideline. For each eye, the number of loci included in the analysis and mean change were reported for each parameter. In addition, the proportion of eyes with a mean change of ≤ –7 dB (i.e., a decline of ≥7 dB) was also reported. Analyses were conducted using IBM SPSS Statistics for Windows, version 29 (IBM Corp).

## Results

From a total of 70 patients with biallelic PLP variants in the *USH2A* gene, 53 (76%) were excluded from analysis due to absent HAR (n = 24, 34%), lack of ≥2 years longitudinal data (n = 23, 33%), an HAR that was larger than the 10-2 grid so no HRS loci were available (n = 4, 6%), and absence of imaging data (n = 2, 3%). Seventeen left eyes from 17 patients (10 women and 7 men) were included in the analysis, with a median (interquartile range) age of 37.0 (24.6–42.9 years) years at baseline (Table 1). The median (interquartile range) age of onset in visual symptoms was 18 (15–24 years) years. OCT scans showed 2 patients with cystoid macular edema, which were stable throughout the study period (patients 2 and 12), 2 patients with optic disc drusen and stable disc elevation (patients 4 and 8), and 2 patients with mild stable epiretinal membrane (patients 5 and 7). Patient 1 had severe photoreceptor loss encroaching into the fovea. The genotypes and protein changes for these 15 patients are shown in Table 1, with genomic coordinates available in Table S2 (available at www.ophthalmologyscience.org). No de novo variants were identified. In most eyes, ultrawide retinal imaging showed presence of bone spicules, speckled hypoautofluorescence in the periphery, and a central HAR (Fig. S2, available at www.ophthalmologyscience.org).

**Table 1:** Demographic, Left-Eye Clinical, and Variant Data of Patients with *USH2A*-Retinopathy

Patient	Sex	Age at Onset, yr	Age at Baseline	Lens	BCVA	Allele 1		Allele 2	
						cDNA	Protein	cDNA	Protein
1∗	F	10	53	IOL	20/250	c.2299del	p.Glu767Serfs∗21	c.7595-2144A>G	p.Lys2532Thrfs56∗
2∗	M	11	13	Clear	20/50	c.9424G>T	p.(Gly3142∗)	c.11864G>A	p.(Trp3955∗)
3∗	M	14	17	Clear	20/25	c.1859G>T	p.(Cys620Phe)	c.12067-2A>G	p.(?)
4∗	F	14	17	Clear	20/25	c.949C>A	p.(Arg317)=	c.1256G>T	p.(Cys419Phe)
5	F	15	39	Clear	20/32	c.2276G>T	p.Cys759Phe	c.13316C>T	p.(Thr4439Ile)
6∗	M	16	37	Mild PSC	20/40	c.2299del	p.Glu767Serfs∗21	c.12697_12698del	p.(Trp4233Aspfs∗4)
7∗	M	16	28	Clear	20/20	c.10561T>C	p.(Trp3521Arg)	c.3086G>T; 9258+1G>A	p.(Gly1029Val); (?)
8∗	F	17	17	Clear	20/25	c.949C>A	p.(Arg317=)	c.1256G>T	p.(Cys419Phe)
9	F	18	39	Mild PSC	20/32	c.2299del	p.Glu767Serfs∗21	c.13335_13347delinsCTTG	p.(Glu4445_Ser4449del insAspLeu)
10	M	21	24	Clear	20/20	c.1256G>T	p.(Cys419Phe)	c.2276G>T	p.Cys759Phe
11	F	21	42	Clear	20/25	c.1679del	p.(Pro560Leufs∗31)	c.10073G>A	p.(Cys3358Tyr)
12∗	F	22	30	Clear	20/63	c.7595-3C>G	p.(?)	c.13942_13943delinsT	p.(Gly4648Tyrfs∗30)
13	M	24	36	Clear	20/25	c.2276G>T	p.Cys759Phe	c.5884del	p.(Arg1962Glufs∗5)
14	F	29	43	Mild PSC	20/32	c.475C>T	p.(Gln159∗)	10073G>A	p.(Cys3358Tyr)
15	M	29	61	Mild PSC	20/25	c.10073G>A	p.(Cys3358Tyr)	c.10073G>A	p.(Cys3358Tyr)
16	F	35	42	Clear	20/20	c.7681G>A	p.(Gly2561Arg)	c.10525A>G	p.(Lys3509Glu)
17	F	43	43	Clear	20/20	c.3812-3_3837dup	p.(Met1280∗)	c.5572+1G>A	p.(?)

BCVA = best-corrected visual acuity; cDNA = coding DNA; F = female; IOL = intraocular lens; M = male; PSC = posterior subcapsular cataract.

∗ Hearing loss at birth (Usher syndrome type 2).

### Baseline Analysis

**Figure 3:**
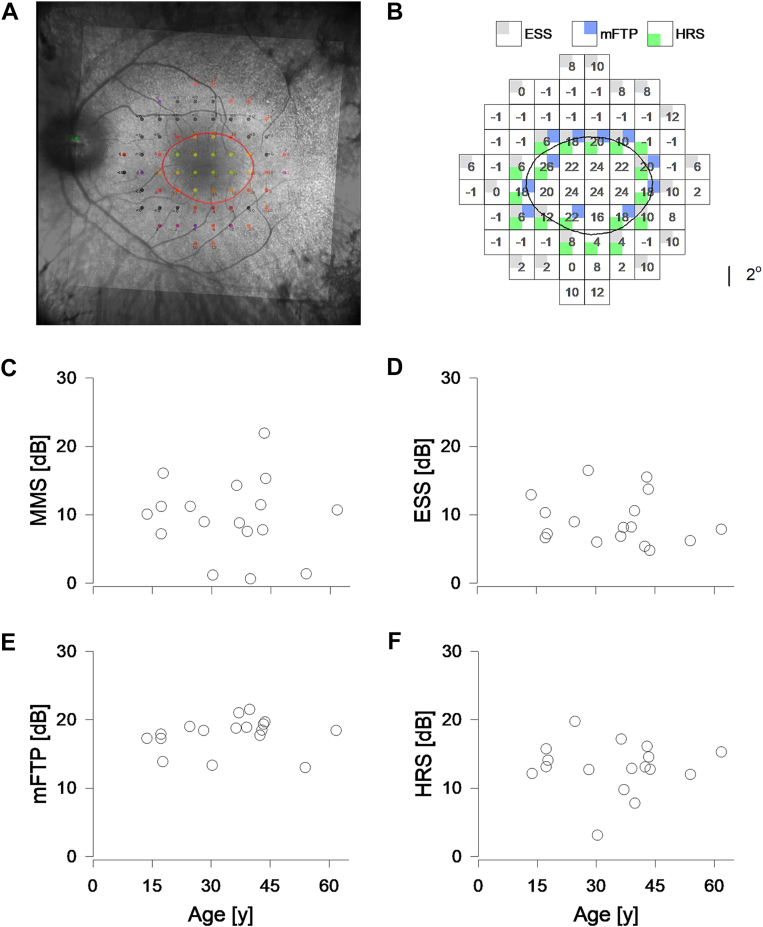
Cross section microperimetry results in *USH2A*-retinopathy. **A,** A representative (patient 5) baseline showing image registration of the hyperautofluorescent ring (red circle) marked from the Spectralis cSLO (inner square, rotated) to the MAIA cSLO. **B,** Using the same eye shown in panel A, each locus of the 10-2 grid is represented by a 2° × 2°, nonoverlapping square with the corresponding retinal sensitivity shown. The AF ring boundary (black line) is superimposed onto the grid. With each locus, gray, blue, and green subsquares indicate loci included in ESS, mFTP, and HRS analysis, respectively. **C–F,** Mean macular sensitivity (C), ESS (D), mFTP (E), and HRS (F) are plotted against age at baseline. AF = autofluorescence; cSLO = confocal scanning laser ophthalmoscope; dB = decibels; ESS = edge of scotoma sensitivity; HRS = hyperautofluorescent ring sensitivity; MAIA = Macular Integrity Assessment; mFTP = modified RUSH2A study-defined functional transitional point; MMS = mean macular sensitivity.

A representative example is shown to demonstrate extraction of all 4 parameters from 1 microperimetry visit (patient 5, Fig. 3). After registration of the MAIA and Spectralis cSLO images (Fig. 3A), the Cartesian boundary of the HAR was extracted and plotted onto the 10-2 grid (Fig. 3B). Mean macular sensitivity (all 68 loci), ESS (gray top left, 25 loci), mFTP (blue top right, 11 loci), and HRS (green bottom left, 17 loci) values were then analyzed at baseline.

In the 17 eyes, baseline mean ± standard deviation MMS, ESS, mFTP, and HRS values were 9.7 ± 5.5 dB, 9.2 ± 3.6 dB, 17.9 ± 2.4 and 13.1 ± 3.8 dB, respectively. Repeated measures ANOVA showed there was an overall significant difference in means (*P* < 0.05), with pairwise comparisons revealing mFTP was significantly higher than the 3 other metrics, due to the ≥8 dB sensitivity baseline criterion. Hyperautofluorescent ring sensitivity was significantly higher than both MMS and ESS but there was no significant difference between MMS and ESS. When analyzed against age, there was minimal linear association between age and MMS (y = –0.03x + 10.95, *R*^*2*^ < 0.01; Fig. 3C), ESS (y = –0.04x + 10.63, *R*^*2*^ = 0.03; Fig. 3D), HRS (y = –0.01x + 13.30 *R*^*2*^ < 0.01; Fig. 3F), or mFTP (y = 0.03x + 16.91, *R*^*2*^ = 0.02; Fig. 3E) at baseline.

### Longitudinal Trend Analysis

**Figure 4:**
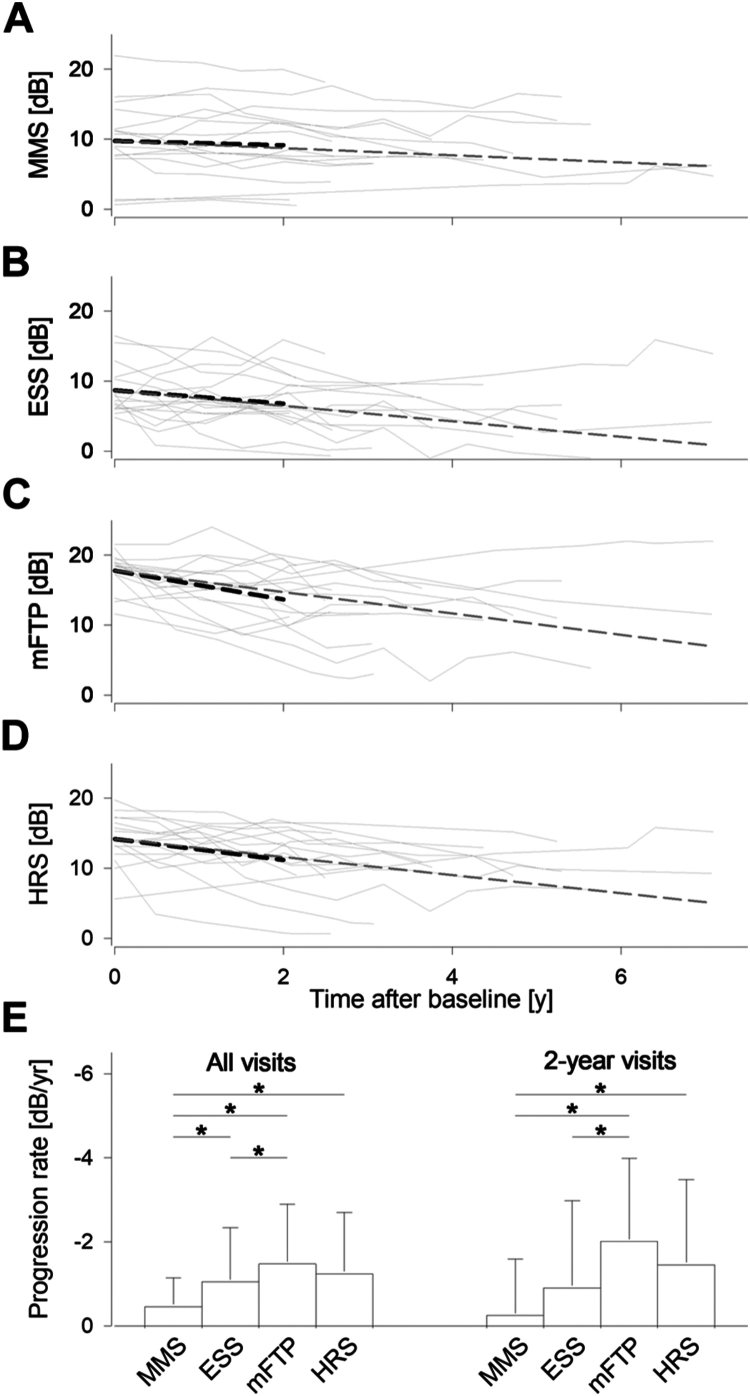
Longitudinal results in *USH2A-*retinopathy. **A–D,** Mean macular sensitivity, ESS, mFTP, and HRS progression is plotted for each eye (solid gray lines). Average progression rate for 2-year (dashed black line) and all (dashed gray line) data are also shown. **E,** Mean MMS, HRS, mFTP, and ESS progression rates across all visits are shown on the left. Two-year progression rates are shown on the right. Error bars represent 1 standard deviation, ∗ denotes statistical significance from pairwise comparisons. dB = decibels; ESS = edge of scotoma sensitivity; HRS = hyperautofluorescent ring sensitivity; mFTP = modified RUSH2A study-defined functional transitional point; MMS = mean macular sensitivity.

Mean macular sensitivity, ESS, mFTP, and HRS loci were defined at baseline and analyzed across subsequent visits (Figure 4A–D). Across all 17 eyes, using all available longitudinal data, the mean follow-up time was 3.99 ± 1.6 years. Repeated measures ANOVA showed significant difference in progression between the 4 parameters, with Bonferroni post hoc tests revealing MMS progression rate (–0.51 ± 0.63 dB/year) was significantly slower than ESS (–1.11 ± 1.23 dB/year), mFTP (–1.53 ± 1.37 dB/year) and HRS (–1.29 ±1.41 dB/year, Fig. 4E, left, all *P* < 0.05). Additionally, mFTP declined significantly faster than ESS (*P* < 0.05). There was no statistical difference between mFTP and HRS (*P* = 0.37).

Two-year progression rates were available for 16 eyes (Fig. 4E, right), due to no data in patient 12. The mean follow-up time was 2.1 ± 0.1 years. Similar to overall progression rate findings, there was a significant difference in progression between the 4 parameters, with pairwise comparison showing MMS (–0.30 ± 1.28 dB/year) rates were slower than HRS (–1.51 ± 1.98 dB/year) and mFTP (–2.06 ± 1.92 dB/year). However, ESS (–0.96 ± 2.02 dB/year) progression rate was not significantly different to MMS (*P* = 0.30). Again, there was no statistical difference between mFTP and HRS (*P* = 0.21).

As MAIA was recorded in most eyes at 6-monthly intervals, a subanalysis was performed at 6, 12, and 18 months to detect significant difference in linear estimates of progression rates between the 4 parameters. At 6 months (n = 12, mean follow-up time 0.53 ± 0.08 year), progression rates were –1.11 ± 2.54 dB/year, –2.62 ± 5.94 dB/year, –4.75 ± 4.59 dB/year, and –3.11 ± 4.93 dB/year for MMS, ESS, mFTP, and HRS, respectively. Repeated measures ANOVA showed significant difference between the progression rates, with Bonferroni post hoc test revealing significant difference only between MMS and mFTP. The findings at 1 year (n = 15, mean follow-up time 1.08 ± 0.07 year) was similar to 6 months, with pairwise comparison detecting significant difference only between MMS (–0.33 ± 1.84 dB/year) and mFTP (–2.91 ± 3.47 dB/year). At 18 months (n = 12, mean follow-up time 1.55 ± 0.11 year), pairwise comparison showed MMS (–0.70 ± 1.71 dB/year) was significantly slower than both mFTP (–2.74 ± 2.75 dB/year) and HRS (–2.52 ± 2.90 dB/year).

### Prespecified Event-Based Analysis

At the final study visit, 16, 11, 16, and 14 eyes had with ≥5 eligible prespecified (i.e., ≥8 dB at baseline + criterion for each respective metric) loci in MMS, ESS, mFTP, and HRS for event-based analyses, respectively (Table 3; Table S4, available at www.ophthalmologyscience.org). The raw number of eyes that showed mean change of ≥7 dB were similar between ESS (5), mFTP (7), and HRS (6), but noticeably less in MMS (2). The proportion of eyes that demonstrated a clinically significant mean change (FDA criteria) was similar between ESS (2-year 36.4% and 4-year 45.5%), mFTP (2-year 33.3% and 4-year 43.8%), and HRS (2-year 28.5% and 4-year 42.9%), but noticeably less in MMS (2-year 13.3% and 4-year 12.5%).

**Table 3:** Summarizing Event-Based Analysis, Including Only Eyes with ≥5 Prespecified Loci (≥8 dB) at Baseline

		2-Yr	All Visits∗
MMS	No. of eyes	15	16
	No. of loci analyzed (mean ± SD, range)	37.8 ± 13.2, 8 to 59	35.9 ± 14.9, 7 to 59
	Mean change (mean ± SD, range [dB])	–2.7 ± 3.4, –9.2 to 3.1	–3.2 ± 3.4, –8.6 to 6.0
	Proportion of eyes with mean change ≤ –7 dB	2/15 = 13.3%	2/16 = 12.5%
	Mean rate (mean ± SD [dB/yr])	–1.2 ± 1.7	–1.0 ± 1.0
ESS	No. of eyes	11	11
	No. of loci analyzed (mean ± SD, range)	12.9 ± 3.9, 6 to 18	12.9 ± 3.9, 6 to 18
	Mean change (mean ± SD, range [dB])	–5.2 ± 5.0, –13.6 to 3.6	–7.3 ± 3.8, –13.6 to –0.88
	Proportion of eyes with mean change ≤ –7 dB	4/11 = 36.4%	5/11 = 45.5%
	Mean rate (mean ± SD [dB/yr])	–2.4 ± 2.4	–2.2 ± 1.5
mFTP	No. of eyes	15	16
	No. of loci analyzed (mean ± SD, range)	9.3 ± 2.9, 6 to 16	9.1 ± 3.0, 6 to 16
	Mean change (mean ± SD, range [dB])	–5.5 ± 4.1, –14.0 to 1.3	–6.4 ± 4.3, –14.3 to 5.0
	Proportion of eyes with mean change ≤ –7 dB	5/15 = 33.3%	7/16 = 43.8%
	Mean rate (mean ± SD [dB/yr])	–2.6 ± 1.9	–1.9 ± 134
HRS	No. of eyes	14	14
	No. of loci analyzed (mean ± SD, range)	16.0 ± 3.9, 11 to 24	16.0 ± 3.9, 11 to 24
	Mean change (mean ± SD, range [dB])	–4.5 ± 4.9, –14.2 to 2.5	–6.8 ± 3.7, –13.6 to –0.6
	Proportion of eyes with mean change ≤ –7 dB	4/14 = 28.5%	6/14 = 42.9%
	Mean rate (mean ± SD [dB/yr])	–2.1 ± 2.3	–2.0 ± 1.5

dB = decibels; ESS = edge of scotoma sensitivity; HRS = hyperautofluorescent ring sensitivity; mFTP = modified RUSH2A study-defined functional transitional point; MMS = mean macular sensitivity; SD = standard deviation.

∗ Follow-up duration [yrs]: MMS 4.1 ± 1.6; ESS 3.8 ± 1.4; FTP 4.1 ± 1.6; HRS 4.0 ± 1.4.

Repeated measures ANOVA showed a significant difference in mean change (MMS –4.6 ± 2.5 dB, ESS –8.0 ± 3.3 dB, mFTP –7.9 ± 3.2 dB, and HRS –7.6 ± 3.7 dB) with pairwise comparisons revealing MMS was significantly lower than the other 3 metrics.

Not surprisingly, the raw number of eyes with a mean change of ≤ –7 dB at 2 years was generally lower than that of the overall data (MMS 2, ESS 4, mFTP 5, and HRS 4).

### Factors Impacting Trend-Based Progression Rates

**Figure 5:**
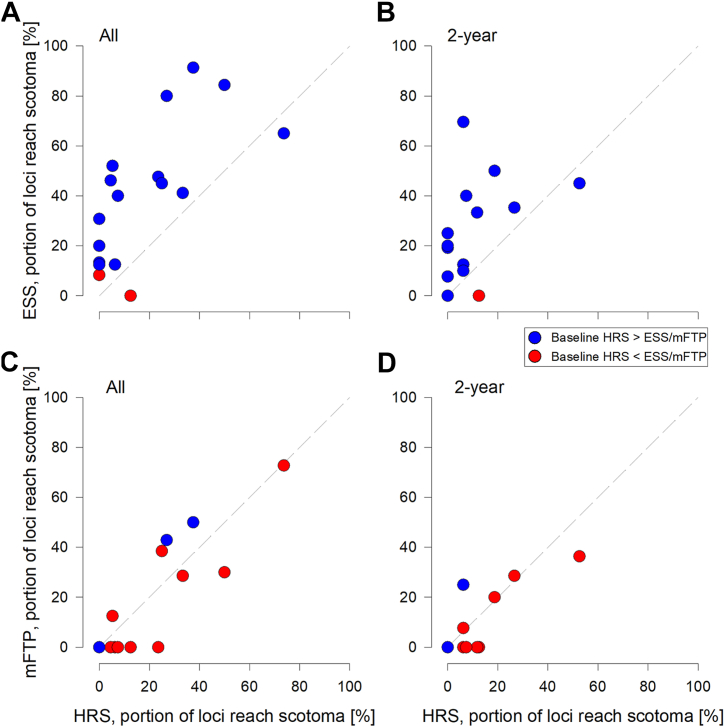
Factors affecting trend-based progression analysis. **A,** In all longitudinal data, in each eye, the proportion of ESS loci that reached scotoma during the observation period is plotted against the proportion of HRS loci that reached scotoma. Blue and red fill show higher HRS and ESS values at baseline, respectively. Gray dashed line indicates the identity line. **B,** The proportion of ESS loci reaching scotoma is plotted against the proportion of HRS loci reaching scotoma within the 2-year observation period. All other details as per panel A. **C, D,** The proportion of ESS loci reaching scotoma plotted against the proportion of mFTP loci reaching scotoma across all (C) and 2-year (D) observation periods. Blue and red fill show higher HRS and mFTP values at baseline, respectively. All other details as per panel A. ESS = edge of scotoma sensitivity; HRS = hyperautofluorescent ring sensitivity; mFTP = modified RUSH2A study-defined functional transitional point.

To further investigate possible reasons for the faster mFTP and HRS decline than ESS in the trend analysis, the percentage of loci that reached scotoma (defined as 2 consecutive –1 dB) in ESS was plotted against percentage of loci that reached scotoma in mFTP and HRS. Examination of ESS versus HRS showed that, in all longitudinal data (Fig. 5A), the majority (15/17) of the data are above the identity line, suggesting that the floor effect was more of a factor in ESS than HRS. Furthermore, at baseline, the mean difference between HRS and ESS (HRS-ESS) was 5.1 ± 3.7 dB, with the majority of eyes showing greater HRS value (15/17, blue). Similar to final visit findings, the 2-year longitudinal data (Fig. 5B) also showed the majority (12/16) of eyes with higher proportion of ESS than HRS loci reaching scotoma during the observation period as well as more eyes with higher HRS baseline value. Floor effect was similar between mFTP and HRS (Fig. 5C, D). However, a greater proportion of eyes showed higher baseline sensitivity in mFTP than HRS (14/17 in both time periods).

## Discussion

In the current study, we showed HRS, which colocalized microperimetry data to an anatomical marker of progression, returned a similar trend-based progression rate as mFTP but a significantly faster rate than MMS. Trend-based analysis showed progression rate was significantly faster than MMS at 0.5 and 1.5 years from baseline for mFTP and HRS, respectively. Additionally, both HRS and mFTP showed comparable proportions of eyes that met the FDA threshold for clinically meaningful change at 2-years and final visit.

In our *USH2A* cohort, baseline MMS using the MAIA microperimeter was 9.7 ± 5.5 dB, which was higher than MMS reported from another *USH2A* cohort (5.9 ± 5.0 dB).^40^ Although MAIA microperimeter was used in both studies, several factors could have contributed to the lower MMS values in the previous study. First, due to the narrower inclusion criteria such as presence of analyzable HAR in the current study, patient age distribution (41% <35 years, 47% 35–45 years, 12% ≥45 years) was younger compared with the previous work (40% <35 years, 36% 35-45 years, 24% ≥45 years). Additionally, the test grid used in the previous study extended further away from the foveal center than the current study (15° versus 10°). Given that the literature has shown that microperimetry retinal sensitivities decrease as a function of age as well as eccentricity,^16^ these 2 factors could possibly contribute to the lower MMS observed in the aforementioned work. In terms of the HAR prevalence, the proportion of *USH2A* patients with analyzable HAR at baseline was (40/70 = 57%, including 23 patients with no longitudinal data), comparable with previously reported (∼60%).^29^^,^^41^

The average MMS progression rates in our study were –0.51 dB/year and –0.30 dB/year for the final 4-year and 2-year data, respectively. To our knowledge, to date, only 1 study in literature has reported microperimetry progression in *USH2A*-retinopathy, with similar MMS progression rates reported by the RUSH2A study (4-year: –0.65 dB/year, 2-year: –0.38 dB/year).^23^ However, direct comparison of progression rates between RUSH2A and the current study is not appropriate due to the difference in test grid patterns, 89 versus 68 loci. Using the same MAIA microperimeter and the 10-2 grid, comparable MMS progression rates have been reported in *RPGR*^42^ and *MYO7A*^43^ associated retinopathy (–0.7 to –0.4 dB/year). To capture loci with a faster rate of progression, we investigated ESS, mFTP and HRS zones. We have previously shown that ESS produced a statistically faster progression in *USH2A*-retinopathy than MMS.^20^ However, given the increased variability in microperimetry data at the border of deep scotoma,^24^ we hypothesized that faster progression rate can be detected if loci were preselected based on colocalization with the HAR, a known anatomic transition zone marking disease progression in RP. Our data demonstrated that HRS progression rates were trending higher than ESS but were not significantly different in repeated measures ANOVA analysis. More importantly, there was no statistical difference between HRS and mFTP in trend analysis. Our data suggest that floor effect and lower baseline sensitivity values contributed to the lower ESS progression rates. One strategy to mitigate retinal sensitivity floor effect is to terminate the rate calculation when scotoma was observed in 2 consecutive visits.^18^

We acknowledge there are several limitations in our study including a single site study and the small sample size due to exclusion of eyes without a HAR within the grid and short follow-up. However, despite of the difference in test grids, our mFTP mean changes (2-year –5.5 dB and 4-year –6.4 dB) were similar to the RUSH2A-FTP progression rates of –6.92 dB over 4 years.^23^ More importantly, the RUSH2A group reported 19% and 46% of their cohort met the FDA threshold of clinically meaningful loss in FTP, similar to the findings of 33.3% and 43.8% in our mFTP analyses at 2- and 4-year timepoints. Our data also showed similar proportions in ESS and HRS compared to mFTP, with noticeably smaller proportion in MMS. However, we note that ESS may not be applicable in RP patients with early disease due to limited scotomatous loci in the 10-2 grid.

In conclusion, we developed an mFTP selection algorithm based on the RUSH2A study, which can be used in future studies that have collected the more commonly used 10-2 MAIA data. The outcome from mFTP analysis is comparable to HRS, adding strength to the anatomical basis for using mFTP as an end point measure. Future multicentre studies analyzing longitudinal 10-2 grid MAIA data can adopt mFTP algorithm to detect a faster disease progression and provide trend-based and event-based natural history data for sample size calculations.

## Supplementary Data

Supplementary Material_clean
