# Supplementary material for: Anatomy- versus Sensitivity-Based Loci Preselection in Detecting USH2A-Retinopathy Microperimetric Progression
Source: Ophthalmol Sci. 2025 Nov 24;6(2):101018. doi: 10.1016/j.xops.2025.101018 (PMC12811453; doi:10.1016/j.xops.2025.101018)
Supplement: Supplementary Material_clean [file mmc1.pdf]

Supplementary Material

Anatomy- versus sensitivity-based loci preselection in detecting *USH2A*-retinopathy microperimetric progression

Jason Charng, David Alonso-Caneiro, Tina M Lamey, Jennifer A Thompson, Jeremiah KH Lim, Elaine Ong, Terri L McLaren, Fred K Chen

Supplementary Figure S1. Pathway schematic for FTP analysis.

Supplementary Figure S2. Ultrawide retinal and fundus autofluorescence images in study eyes at baseline

Supplementary Table S2. Genomic coordinates (Build GRCh37) for *USH2A* variants described in this study

Supplementary Table S4. Number of prespecified loci and in each eye for MMS, ESS, mFTP and HRS metrics

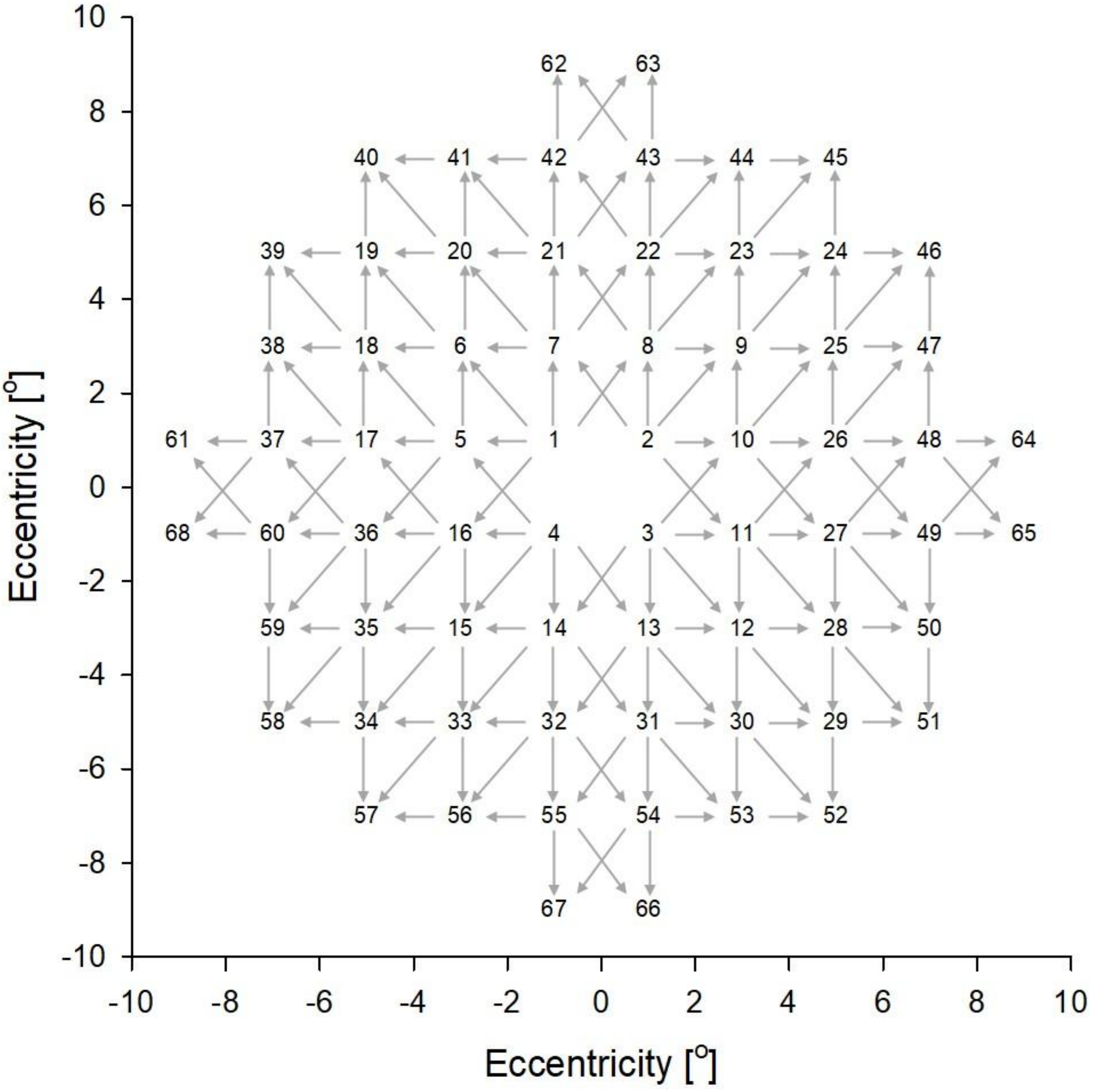

Supplementary Figure S1. Pathway schematic for FTP analysis. For each locus, the peripheral adjacent loci (ranging from 5 centrally and 2 peripherally) utilized to calculate retinal sensitivity change are indicated by the arrows. The numbers on the grid correspond to loci ID in the accompanied Excel Spreadsheet.

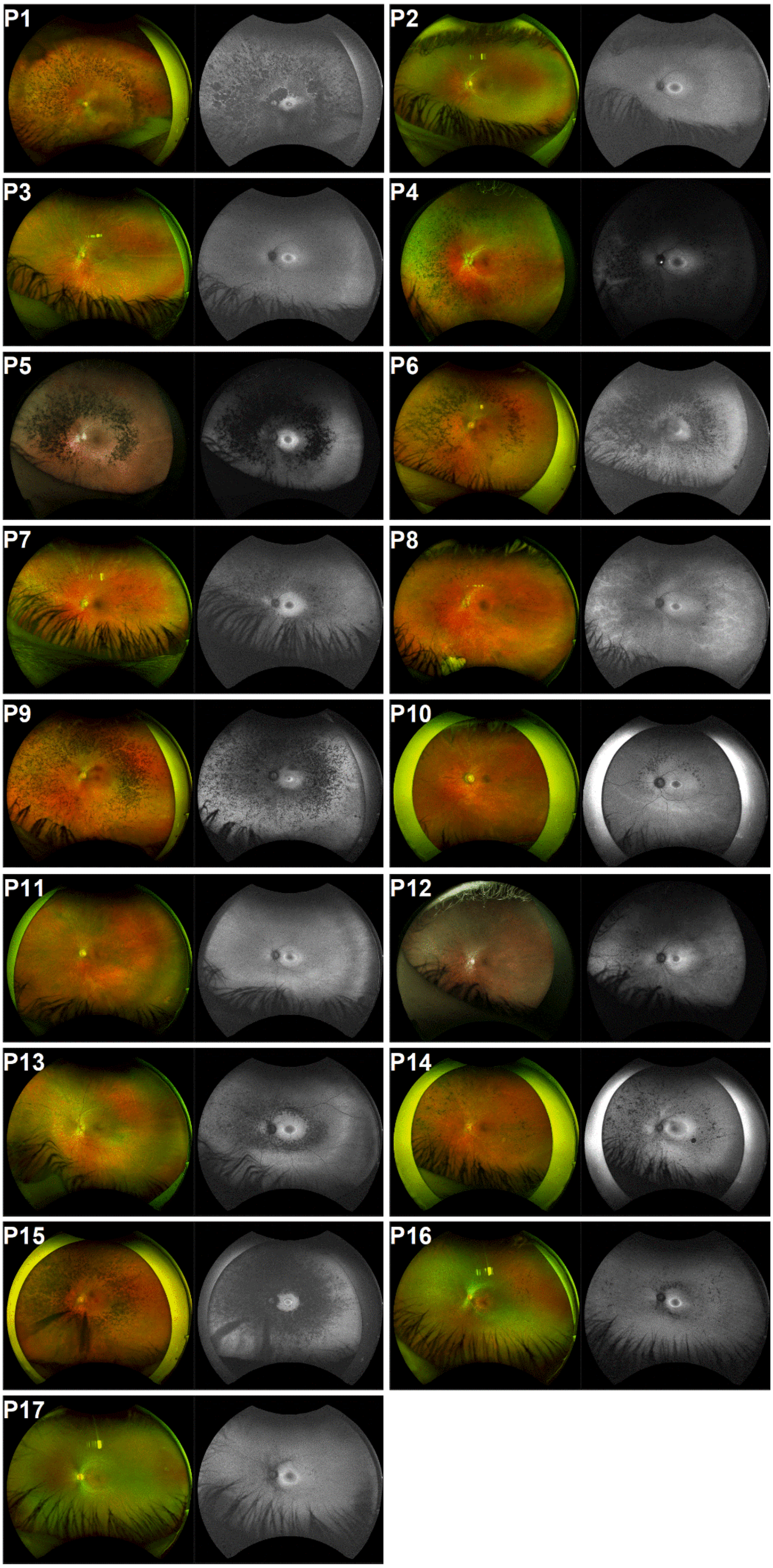

Supplementary Figure S2. Ultrawide retinal (left) and fundus autofluorescence (right) images in study eyes at baseline

Supplementary Table S2: Genomic coordinates (Build GRCh37) for *USH2A* variants described in this study in accordance with coding and genomic reference sequences, NM_206933.4 and NC_000001.10, respectively

**Coding variant**	**Genomic variant (GRCh37)**
c.475C>T	Chr1:g.216595204G>A
c.949C>A	g.216498841G>T
c.1256G>T	g.216497582C>A
c.1679del	g.216465677AG>A
c.1859G>T	g.216462734C>A
c.2276G>T	g.216420460C>A
c.2299del	g.216420437del
c.3086G>T	g.216390800C>A
c.3812-3_3837dup	g.216371902_216371930dup
c.5572+1G>A	g.216251430C>T
c.5884del	g.216070265CT>C
c.7595-2144A>G	g.216064540T>C
c.7595-3C>G	g.216062399G>C
c.7681G>A	g.216062310C>T
c.9258+1G>A	g.216017635C>T
c.9424G>T	g.215990485C>A
c.10073G>A	g.215963510C>T
c.10561T>C	g.215956104A>G
c.11864G>A	g.215901574C>T
c.12067-2A>G	g.215853720T>C
c.12697_12698del	g.215848555_215848556del
c.13316C>T	g.215847937G>A
c.13335_13347delinsCTTG	g.215847906_215847918delinsCAAG
c.13942_13943delinsT	g.215844504CC>A

Supplementary Table S4: Number of prespecified loci and in each eye for MMS, ESS, mFTP and HRS using all longitudinal data. A prespecified locus is defined by retinal sensitivity of 8 dB or more at baseline combined with the requirement of each metric.

	**MMS**	**ESS**	**mFTP**	**HRS**
Pid	Total prespecified loci	Total prespecified loci	Total prespecified loci	Total prespecified loci
1	8	8	7	3
2	35	16	11	16
3	37	9	8	18
4	59	3	7	24
5	32	18	9	12
6	34	17	10	11
7	29	15	14	14
8	28	12	8	12
9	4	3	4	3
10	36	13	16	16
11	40	4	7	19
12	7	4	6	3
13	50	12	8	23
14	59	6	9	14
15	44	3	7	13
16	27	16	13	15
17	49	0	6	17
Mean (all)	34.0	9.4	8.8	13.7
Standard deviation	16.4	5.9	3.1	6.2
No. eyes ≥ 5 loci	16	11	16	14
Mean (only eyes ≥ 5 loci)	35.9	12.9	9.1	16.0
Standard deviation	14.9	3.9	3.0	3.9

MMS: average sensitivity of all loci. Edge of scotoma sensitivity

ESS: average sensitivity of all loci adjacent to a scotomatous loci at baseline.

FTP: selection based on a ranking of the proportion peripheral adjacent loci that showed ≥7 dB decrease.

HRS: average sensitivity of stimulus squares which the HAR boundary transects into.
